# PGAdb-builder: A web service tool for creating pan-genome allele database for molecular fine typing

**DOI:** 10.1038/srep36213

**Published:** 2016-11-08

**Authors:** Yen-Yi Liu, Chien-Shun Chiou, Chih-Chieh Chen

**Affiliations:** 1 Central Regional Laboratory, Center for Diagnostics and Vaccine Development, Centers for Disease Control, Taichung 40855, Taiwan; 2 Institute of Medical Science and Technology, National Sun Yat-sen University, Kaohsiung 80424, Taiwan; 3 Medical Science and Technology Center, National Sun Yat-sen University, Kaohsiung 80424, Taiwan

## Abstract

With the advance of next generation sequencing techniques, whole genome sequencing (WGS) is expected to become the optimal method for molecular subtyping of bacterial isolates. To use WGS as a general subtyping method for disease outbreak investigation and surveillance, the layout of WGS-based typing must be comparable among laboratories. Whole genome multilocus sequence typing (wgMLST) is an approach that achieves this requirement. To apply wgMLST as a standard subtyping approach, a pan-genome allele database (PGAdb) for the population of a bacterial organism must first be established. We present a free web service tool, PGAdb-builder (http://wgmlstdb.imst.nsysu.edu.tw), for the construction of bacterial PGAdb. The effectiveness of PGAdb-builder was tested by constructing a pan-genome allele database for *Salmonella enterica* serovar Typhimurium, with the database being applied to create a wgMLST tree for a panel of epidemiologically well-characterized *S*. Typhimurium isolates. The performance of the wgMLST-based approach was as high as that of the SNP-based approach in Leekitcharoenphon’s study used for discerning among epidemiologically related and non-related isolates.

Molecular subtyping of bacterial isolates has been fundamental for epidemiologic study of infectious diseases. Subtyping methods used for disease outbreak investigation and surveillance across regions and countries must be standardized so that the results can be compared across laboratories. For example, pulsed-field gel electrophoresis (PFGE) is a good example; it has been standardized and successfully implemented as a common subtyping tool in the foodborne disease surveillance network—PulseNet^1^. Although PFGE is highly discriminatory to most bacterial organisms, it is labor- and time-consuming and sometimes insufficient in discerning among strains of highly clonal organisms. A multilocus variable-number tandem repeat analysis (MLVA) exhibits a much higher level of discrimination than PFGE in discerning among very closely related strains; however, MLVA is very organism-specific, and comparing its results across laboratories is difficult^2^,^3^. With the advance of next-generation sequencing (NGS) techniques, whole genome sequencing (WGS) has become a practical and powerful subtyping tool for disease outbreak detection^4^,^5^.

To use WGS as a standard subtyping tool for disease surveillance and the investigation of common outbreaks across regions or countries, the layout of fingerprints (genotypes) generated from WGS data must be comparable among laboratories. Currently, NGS platforms generally produce millions of short sequences (reads) for a bacterial strain. The millions of reads can be further assembled into longer sequences (contigs) and annotated using various assemblers^6^,^7^,^8^. A number of algorithms and approaches have been developed for analyzing WGS data^9^,^10^,^11^,^12^,^13^,^14^. Single nucleotide polymorphism (SNP) is an approach frequently used to analyze WGS data for evolutionary study and disease outbreak investigation^15^,^16^,^17^. To apply the SNP approach, a reference genome sequence is required for selecting SNPs from WGS data of strains. When different reference sequences are used, different SNP sets are generally yielded, making the SNP profiles incomparable across laboratories. Whole genome multilocus sequence typing (wgMLST)^14^,^18^, an extended concept of the traditional MLST^19^, is considered an ideal approach to sort out WGS data and generate genetic layouts that are portable and comparable among laboratories. To use wgMLST as a standard subtyping tool, a pan-genome allele database (PGAdb) for the population of a bacterial organism must first be established. In a PGAdb, genes (loci) and their sequence variants (alleles) are designated using a standardized numbering system. An allelic sequence consists of a series of digital numbers and can be portable and comparable across laboratories.

We present a web service tool, PGAdb-builder that can be used for the construction of bacterial pan-genome allele databases. In this paper, we demonstrate the function of the PGAdb-builder by constructing a *S*. Typhimurium PGAdb and generating a wgMLST tree for a panel of epidemiologically well-characterized *S*. Typhimurium isolates, which were sequenced previously by the DTU Food^20^.

## Methods and Implementation

**Figure 1:**
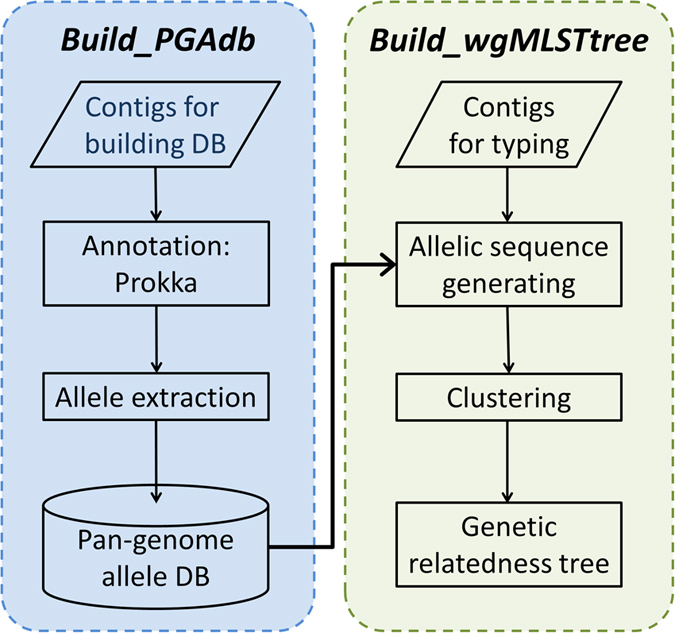
The schematic work flow of PGAdb-builder.

The flowchart for the proposed PGAdb-builder is illustrated in Fig. 1. The PGAdb-builder server comprises two functional modules: *Build_PGAdb* for creating a PGAdb database and *Build_wgMLSTtree* for constructing a wgMLST tree from uploaded genome contigs and formulating genetic relatedness trees by using the PGAdb for generating allelic sequences. The details of the *Build_PGAdb* and *Build_wgMLSTtree* modules are described herein.

### Build_PGAdb

The *Build_PGAdb* module executes the annotation of uploaded genome contigs by using the Prokka pipeline^21^, a rapid bacterial genome annotation tool. Subsequently, the output gff file created in the annotation process is processed to place proteins into orthologous clusters by using the Roary pipeline^22^, a tool that can rapidly process a large-scale collection of genomes. In this module, paralogous genes are excluded from a pan-genome allele dataset. Each orthologous cluster consists of a protein family with 95% (adjustable between 90% and 99%) sequence identity. Each protein family is defined as a locus (gene). The orthologous proteins in each cluster are converted to nucleotide sequences through inference to the ffn file created in the annotation process to establish a pan-genome allele dataset. In this step, sequences in a locus with one or more mismatched nucleotides between each other are defined as different alleles. The loci of a pan-genome allele dataset are then encoded with a prefix string of three alphabetic letters followed by an eight digits serial number (e.g., SAL00000001, SAL00000002…) and the alleles in each locus are simply assigned by a series of integers beginning from 1 to n (e.g. 1, 2, 3, … n).

### Build_wgMLSTtree

The *Build_wgMLSTtree* module compares the uploaded genome contigs of strains by using a PGAdb database and constructs genetic relatedness trees (wgMLST trees). To create a wgMLST tree, the uploaded genome contigs is compared with the built PGAdb using BLASTN^23^. If an allele is present in a locus, the predefined allele number is assigned; however, if an allele is absent, “0” is assigned. After the allele finding process is finished, an “allelic sequence” for an uploaded genome is created. A dendrogram with bootstrap values, which is calculated by the ETE tool kit^24^, is then constructed from allelic sequences with the PHYLIP program^25^ through use of UPGMA clustering algorithm.

### Implementation

The PGAdb-builder server is created through an integration of the *Build_PGAdb* and *Build_wgMLSTtree* modules in PHP scripts. The web page was constructed using HTML, javascript, and PHP. The server runs on a Linux cluster with 2.40 GHz Intel Xeon processors comprising 24 cores. Dendrograms labeled with bootstrap values made using *Build_wgMLSTtree* module are output in the webpage and in a downloadable Newick and a pdf format.

## Webserver

### Input format

**Figure 2:**
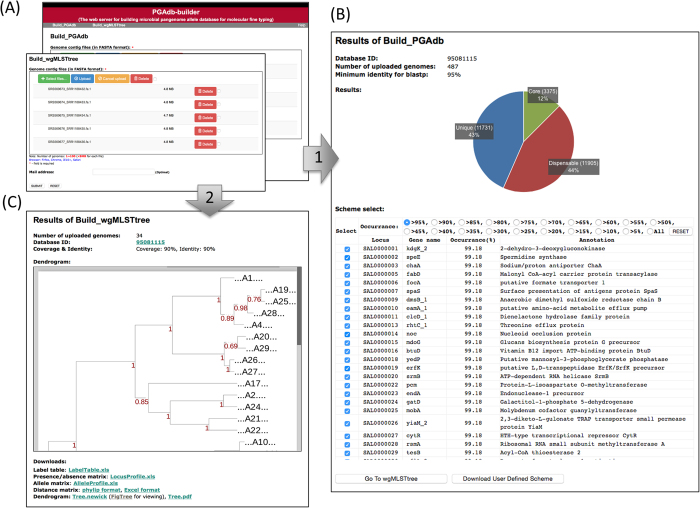
The features of the PGAdb-builder server. (**A**) Input page of the *Build_PGAdb* (right panel) and the *Build_wgMLSTtree* (left panel). (**B**) Output page of the *Build_PGAdb*. (**C**) Output page of the *Build_wgMLSTtree*.

The two modules of PGAdb-builder accepts genome contigs in the FASTA format (Fig. 2A). When the default parameter was usded (protein sequence identity = 95%), *Build_PGAdb* required approcimately 19 hours to construct a database in a test using 487 *S*. Typhimurium genomes (487ST_set). *Build_PGAdb* creates a Database ID after the process finished. *Build_wgMLSTtree* required 4.5 hours to construct a wgMLST tree (with pan-genome scheme) for 34 *S*. Typhimurium genomes by using the PGAdb when the default parameters (alignment coverage ≥ 90%; alignment identity ≥ 90%) were set. Users are encouraged to provide e-mail addresses through which to receive a notification for when a job finishes.

### Output format

The output of *Build_PGAdb* comprises (A) a summary of settings; (B) a pie chart illustrating the numbers (percentages) of loci for the core genome, dispensable genome, and unique genes in the PGAdb; (C) a checkbox menu for the selection of the user-defined scheme; and (D) buttons, to perform “Go To wgMLSTtree” and “Download User Defined Scheme.” The file of the user-defined scheme can be used as the input for the module of *Build_wgMLSTtree* from the “Upload User Defined Scheme” option. Through this mechanism, users can exchange their pan-genome database by sharing their scheme files. The output of *Build_wgMLSTtree* includes (A) a summary of settings, (B) a genetic relatedness tree constructed using the scheme, which is selected by users, and (C) a summary of output files to download. Examples of *Build_PGAdb* and *Build_wgMLSTtree* outputs are shown in Fig. 2B,C, respectively.

## Example Analysis

**Figure 3:**
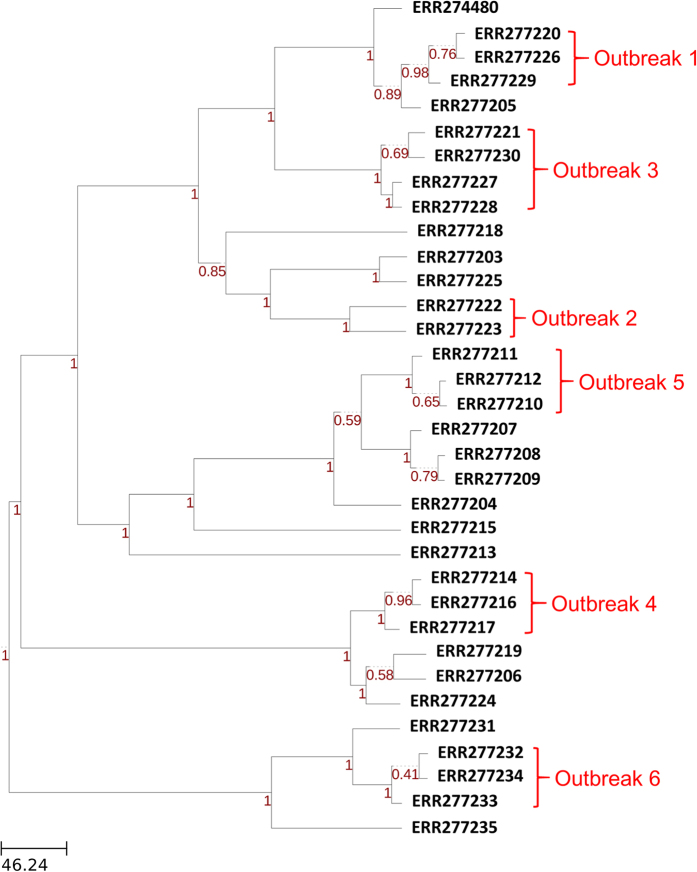
Dendrogram (genetic relatedness tree) for 34 epidemiologically well-characterized *S*. Typhimurium isolates sequenced by DTU Food^20^. Isolates for 6 foodborne disease outbreaks are marked.

We tested the ability of the *Build_PGAdb* module to construct a PGAdb by using 487 *Salmonella* Typhimurium (487ST_set) strains of genome contigs (Table S1), which were downloaded from the National Center for Biotechnology Information (NCBI) Genome database (https://www.ncbi.nlm.nih.gov/genome). The operation required approximately 19 hours on a Linux server with 2.40 GHz Intel Xeon processors comprising 24 cores. The *S.* Typhimurium PGAdb contained 27,011 loci, of which 12.5% (3,375 loci) belonged to the core genome, 44% (11,905 loci) belonged to the dispensable genome, and 43.5% (11,731 loci) belonged to the unique genes. In this step, we defined the core genome as having genes present in 95% of the tested genomes, a dispensable genome as having genes present in two or more but less than 95% of the genomes, and unique genes as being present only in a single genome. The PGAdb from the 487ST_set was then used to construct a wgMLST tree for 34 epidemiologically well-characterized *S*. Typhimurium isolates by using the *Build_wgMLSTtree* module. The allelic sequences for the 34 isolates were formed on the basis of the 27,011 loci for the core genome. As illustrated in Fig. 3, the genetic relationships among the 34 isolates constructed using the wgMLST-based approach were highly concordant with the relationships of the isolates determined using the SNP-based method, as shown in a previous study^20^.

## Conclusion

The proposed online tool PGAdb-builder, comprising two modules, *Build_PGAdb* and *Build_wgMLSTtree*, was established to enable users to use WGS data to construct bacterial pan-genome allele databases and to apply the databases to create genetic relatedness trees for bacterial strains. A strong advantage of the PGAdb-builder server is that the built PGAdb with the user-defined scheme can be reused through uploading the downloaded “User defined scheme file” (UDS file), which records the database ID and the defined scheme. Through this mechanism, users can exchange their PGAdbs by only sharing the UDS files. This PGAdb-builder would be a useful online tool for the construction of bacterial pan-genome allele databases and construction of genetic relatedness tree.

## Additional Information

**How to cite this article**: Liu, Y.-Y. *et al*. PGAdb-builder: A web service tool for creating pan-genome allele database for molecular fine typing. *Sci. Rep.* **6**, 36213; doi: 10.1038/srep36213 (2016).

## Supplementary Material

Supplementary Information
