# Supplementary material for: PGAdb-builder: A web service tool for creating pan-genome allele database for molecular fine typing
Source: Sci Rep. 2016 Nov 8;6:36213. doi: 10.1038/srep36213 (PMC5099940; doi:10.1038/srep36213)
Supplement: Supplementary Information [file srep36213-s1.pdf]

PGAdb-builder: The web server for building microbial whole-genome multi-locus sequence typing database for molecular fine typing

Yen-Yi Liu [1]#. Chien-Shun Chiou [1]# and Chih-Chieh Chen [2,3]*

Table S1. Accession numbers of 487 *Salmonella enterica* serovar Typhimurium genomes used for testing the operation of Build_PGAdb module of PGAdb-builder

GCA_000006945.1	GCA_000493675.1	GCA_001034525.1	GCA_001153005.1
GCA_000022165.1	GCA_000495135.2	GCA_001034545.1	GCA_001153825.1
GCA_000027025.1	GCA_000495155.2	GCA_001034565.1	GCA_001154285.1
GCA_000187785.2	GCA_000495175.2	GCA_001034585.1	GCA_001154585.1
GCA_000188735.1	GCA_000495195.2	GCA_001034595.1	GCA_001155485.1
GCA_000210855.2	GCA_000495215.2	GCA_001034605.1	GCA_001159405.1
GCA_000213635.1	GCA_000495235.1	GCA_001034615.1	GCA_001161925.1
GCA_000252875.1	GCA_000505085.1	GCA_001038035.1	GCA_001162405.1
GCA_000283735.1	GCA_000505505.1	GCA_001038085.1	GCA_001164125.1
GCA_000292755.1	GCA_000614485.1	GCA_001038095.1	GCA_001164765.1
GCA_000312745.2	GCA_000636135.1	GCA_001038105.1	GCA_001172145.1
GCA_000312765.2	GCA_000648415.1	GCA_001038115.1	GCA_001172545.1
GCA_000312785.2	GCA_000648435.1	GCA_001038165.1	GCA_001187325.1
GCA_000312805.2	GCA_000648455.1	GCA_001042425.1	GCA_001187375.1
GCA_000312825.2	GCA_000648475.1	GCA_001085145.1	GCA_001214325.1
GCA_000312845.2	GCA_000648495.1	GCA_001085685.1	GCA_001214365.1
GCA_000312865.2	GCA_000715155.2	GCA_001091445.1	GCA_001214425.1
GCA_000312885.2	GCA_000743055.1	GCA_001093545.1	GCA_001214485.1
GCA_000312905.2	GCA_000784215.1	GCA_001095425.1	GCA_001214545.1
GCA_000314915.1	GCA_000784225.1	GCA_001096565.1	GCA_001214705.1
GCA_000319795.2	GCA_000784235.1	GCA_001097045.1	GCA_001214745.1
GCA_000336155.1	GCA_000784245.1	GCA_001102665.1	GCA_001214765.1
GCA_000336195.1	GCA_000784295.1	GCA_001105145.1	GCA_001214785.1
GCA_000336215.1	GCA_000784315.1	GCA_001110745.1	GCA_001214865.1
GCA_000380325.1	GCA_000786025.1	GCA_001111905.1	GCA_001214965.1
GCA_000430145.3	GCA_000828595.1	GCA_001116925.1	GCA_001215105.1
GCA_000444835.1	GCA_000941015.1	GCA_001119985.1	GCA_001215145.1
GCA_000465175.1	GCA_000973645.1	GCA_001120065.1	GCA_001215365.1
GCA_000465195.1	GCA_000973845.1	GCA_001120665.1	GCA_001215405.1
GCA_000468255.1	GCA_000973885.1	GCA_001125725.1	GCA_001215505.1
GCA_000474335.1	GCA_000973915.1	GCA_001127885.1	GCA_001215525.1
GCA_000474355.1	GCA_000974015.1	GCA_001129385.1	GCA_001215545.1
GCA_000474395.1	GCA_000974095.1	GCA_001130025.1	GCA_001215585.1
GCA_000474475.1	GCA_000974125.1	GCA_001130925.1	GCA_001215665.1
GCA_000474495.1	GCA_000974215.1	GCA_001131525.1	GCA_001215685.1
GCA_000474555.1	GCA_000993725.1	GCA_001135785.1	GCA_001215725.1
GCA_000474575.1	GCA_001026705.1	GCA_001137885.1	GCA_001215765.1
GCA_000486105.1	GCA_001027585.1	GCA_001139325.1	GCA_001215785.1
GCA_000486125.1	GCA_001027595.1	GCA_001140725.1	GCA_001215865.1
GCA_000486345.1	GCA_001027605.1	GCA_001144305.1	GCA_001215885.1
GCA_000493535.1	GCA_001027615.1	GCA_001146465.1	GCA_001215925.1
GCA_001215985.1	GCA_001218065.1	GCA_001219265.1	GCA_001220525.1
GCA_001216085.1	GCA_001218085.1	GCA_001219285.1	GCA_001220545.1

GCA_001216125.1	GCA_001218105.1	GCA_001219305.1	GCA_001220585.1
GCA_001216205.1	GCA_001218125.1	GCA_001219365.1	GCA_001220625.1
GCA_001216345.1	GCA_001218145.1	GCA_001219405.1	GCA_001220645.1
GCA_001216385.1	GCA_001218225.1	GCA_001219425.1	GCA_001220665.1
GCA_001216425.1	GCA_001218245.1	GCA_001219445.1	GCA_001220685.1
GCA_001216445.1	GCA_001218265.1	GCA_001219465.1	GCA_001220705.1
GCA_001216465.1	GCA_001218285.1	GCA_001219485.1	GCA_001220725.1
GCA_001216485.1	GCA_001218305.1	GCA_001219505.1	GCA_001220785.1
GCA_001216545.1	GCA_001218325.1	GCA_001219525.1	GCA_001220865.1
GCA_001216565.1	GCA_001218345.1	GCA_001219545.1	GCA_001220885.1
GCA_001216585.1	GCA_001218365.1	GCA_001219565.1	GCA_001220905.1
GCA_001216605.1	GCA_001218385.1	GCA_001219585.1	GCA_001220925.1
GCA_001216645.1	GCA_001218405.1	GCA_001219645.1	GCA_001220945.1
GCA_001216705.1	GCA_001218425.1	GCA_001219665.1	GCA_001220965.1
GCA_001216765.1	GCA_001218445.1	GCA_001219685.1	GCA_001220985.1
GCA_001216785.1	GCA_001218465.1	GCA_001219705.1	GCA_001221005.1
GCA_001216845.1	GCA_001218505.1	GCA_001219725.1	GCA_001221025.1
GCA_001216985.1	GCA_001218545.1	GCA_001219765.1	GCA_001221045.1
GCA_001217025.1	GCA_001218565.1	GCA_001219805.1	GCA_001221065.1
GCA_001217045.1	GCA_001218585.1	GCA_001219825.1	GCA_001221105.1
GCA_001217085.1	GCA_001218605.1	GCA_001219845.1	GCA_001221125.1
GCA_001217205.1	GCA_001218625.1	GCA_001219865.1	GCA_001221185.1
GCA_001217245.1	GCA_001218665.1	GCA_001219885.1	GCA_001221205.1
GCA_001217265.1	GCA_001218685.1	GCA_001219905.1	GCA_001221225.1
GCA_001217305.1	GCA_001218725.1	GCA_001219925.1	GCA_001221245.1
GCA_001217345.1	GCA_001218745.1	GCA_001219945.1	GCA_001221265.1
GCA_001217385.1	GCA_001218765.1	GCA_001219965.1	GCA_001221285.1
GCA_001217425.1	GCA_001218785.1	GCA_001219985.1	GCA_001221305.1
GCA_001217445.1	GCA_001218805.1	GCA_001220025.1	GCA_001221325.1
GCA_001217545.1	GCA_001218825.1	GCA_001220065.1	GCA_001221345.1
GCA_001217585.1	GCA_001218845.1	GCA_001220105.1	GCA_001221365.1
GCA_001217605.1	GCA_001218865.1	GCA_001220125.1	GCA_001221385.1
GCA_001217625.1	GCA_001218885.1	GCA_001220165.1	GCA_001221405.1
GCA_001217645.1	GCA_001218925.1	GCA_001220205.1	GCA_001221425.1
GCA_001217665.1	GCA_001218965.1	GCA_001220225.1	GCA_001221445.1
GCA_001217705.1	GCA_001218985.1	GCA_001220245.1	GCA_001221485.1
GCA_001217765.1	GCA_001219005.1	GCA_001220265.1	GCA_001221505.1
GCA_001217785.1	GCA_001219025.1	GCA_001220285.1	GCA_001221545.1
GCA_001217825.1	GCA_001219045.1	GCA_001220305.1	GCA_001221565.1
GCA_001217845.1	GCA_001219065.1	GCA_001220325.1	GCA_001221585.1
GCA_001217865.1	GCA_001219085.1	GCA_001220345.1	GCA_001221605.1
GCA_001217885.1	GCA_001219105.1	GCA_001220365.1	GCA_001221625.1
GCA_001217905.1	GCA_001219125.1	GCA_001220385.1	GCA_001221645.1
GCA_001217925.1	GCA_001219145.1	GCA_001220405.1	GCA_001221665.1
GCA_001217945.1	GCA_001219165.1	GCA_001220425.1	GCA_001221685.1
GCA_001217965.1	GCA_001219185.1	GCA_001220445.1	GCA_001221705.1
GCA_001217985.1	GCA_001219205.1	GCA_001220465.1	GCA_001221725.1
GCA_001218025.1	GCA_001219225.1	GCA_001220505.1	GCA_001221745.1
GCA_001221765.1	GCA_001223165.1	GCA_001244105.1	GCA_001247185.1
GCA_001221785.1	GCA_001223185.1	GCA_001244255.1	GCA_001247205.1
GCA_001221805.1	GCA_001223205.1	GCA_001244285.1	GCA_001247265.1
GCA_001221845.1	GCA_001223225.1	GCA_001244335.1	GCA_001247275.1

GCA_001221885.1
GCA_001221905.1
GCA_001221925.1
GCA_001221945.1
GCA_001221985.1
GCA_001222005.1
GCA_001222025.1
GCA_001222065.1
GCA_001222085.1
GCA_001222125.1
GCA_001222145.1
GCA_001222165.1
GCA_001222185.1
GCA_001222205.1
GCA_001222225.1
GCA_001222245.1
GCA_001222325.1
GCA_001222345.1
GCA_001222365.1
GCA_001222385.1
GCA_001222405.1
GCA_001222465.1
GCA_001222485.1
GCA_001222505.1
GCA_001222525.1
GCA_001222565.1
GCA_001222585.1
GCA_001222605.1
GCA_001222625.1
GCA_001222645.1
GCA_001222665.1
GCA_001222705.1
GCA_001222725.1
GCA_001222765.1
GCA_001222785.1
GCA_001222825.1
GCA_001222865.1
GCA_001222885.1
GCA_001222905.1
GCA_001222945.1
GCA_001222965.1
GCA_001223025.1
GCA_001223045.1
GCA_001223085.1
GCA_001223105.1
GCA_001223125.1
GCA_001246265.1
GCA_001246565.1
GCA_001246625.1
GCA_001246695.1
GCA_001246705.1
GCA_001246875.1
GCA_001223245.1
GCA_001223265.1
GCA_001223285.1
GCA_001223305.1
GCA_001223325.1
GCA_001223345.1
GCA_001223365.1
GCA_001223405.1
GCA_001223445.1
GCA_001223465.1
GCA_001223505.1
GCA_001223525.1
GCA_001223545.1
GCA_001240475.1
GCA_001240785.1
GCA_001240805.1
GCA_001240825.1
GCA_001240925.1
GCA_001240935.1
GCA_001240965.1
GCA_001240975.1
GCA_001241075.1
GCA_001241165.1
GCA_001241425.1
GCA_001241505.1
GCA_001241515.1
GCA_001241555.1
GCA_001241585.1
GCA_001241605.1
GCA_001241895.1
GCA_001241905.1
GCA_001241945.1
GCA_001241975.1
GCA_001242275.1
GCA_001242345.1
GCA_001242395.1
GCA_001242565.1
GCA_001243095.1
GCA_001243105.1
GCA_001243255.1
GCA_001243395.1
GCA_001243425.1
GCA_001243505.1
GCA_001243555.1
GCA_001243695.1
GCA_001243815.1
GCA_001245125.1
GCA_001245415.1
GCA_001245495.1
GCA_001245535.1
GCA_001245645.1
GCA_001246225.1
GCA_001244385.1
GCA_001244695.1
GCA_001244705.1
